# The Mechanism of Low-Temperature Oxidation of Carbon Monoxide by Oxygen over the PdCl_2_–CuCl_2_/γ-Al_2_O_3_ Nanocatalyst

**DOI:** 10.3390/nano8040217

**Published:** 2018-04-03

**Authors:** Lev Bruk, Denis Titov, Alexander Ustyugov, Yan Zubavichus, Valeriya Chernikova, Olga Tkachenko, Leonid Kustov, Vadim Murzin, Irina Oshanina, Oleg Temkin

**Affiliations:** 1 Moscow Technological University, Institute of Fine Chemical Technology, Department of General Chemical Technology, Moscow 119571, Russia; lgbruk@mail.ru (L.B.); Denisish26@yandex.ru (D.T.); ustyugov.alexandr@mail.ru (A.U.); oshanina@mirea.ru (I.O.); olegtemkin@mail.ru (O.T.); 2 National Research Centre “Kurchatov Institute”, Moscow 123182, Russia; Zubavichus_YV@nrcki.ru; 3 Functional Materials Design, Discovery and Development Research Group (FMD3), Advanced Membranes and Porous Materials Center (AMPMC), Division of Physical Sciences and Engineering (PSE), King Abdullah University of Science and Technology (KAUST), Thuwal 23955-6900, Kingdom of Saudi Arabia; valeriya.chernikova@kaust.edu.sa; 4 N.D. Zelinsky Institute of Organic Chemistry, Russian Academy of Sciences, Moscow 119991, Russia; ot@ioc.ac.ru; 5 Deutsches Elektronen-Synchrotron DESY, D-22607 Hamburg, Germany; vadim.murzin@gmail.com

**Keywords:** carbon monoxide, palladium, copper, nanocatalyst, oxidation

## Abstract

The state of palladium and copper on the surface of the PdCl_2_–CuCl_2_/γ-Al_2_O_3_ nanocatalyst for the low-temperature oxidation of CO by molecular oxygen was studied by various spectroscopic techniques. Using X-ray absorption spectroscopy (XAS), powder X-ray diffraction (XRD), and diffuse reflectance infrared Fourier transform spectroscopy (DRIFTS), freshly prepared samples of the catalyst were studied. The same samples were also evaluated after interaction with CO, O_2_, and H_2_O vapor in various combinations. It was shown that copper exists in the form of Cu_2_Cl(OH)_3_ (paratacamite) nanophase on the surface of the catalyst. No palladium-containing crystalline phases were identified. Palladium coordination initially is comprised of four chlorine atoms. It was shown by XAS that this catalyst is not capable of oxidizing CO at room temperature in the absence of H_2_O and O_2_ over 12 h. Copper(II) and palladium(II) are reduced to Cu(I) and Pd(I,0) species, respectively, in the presence of CO and H_2_O vapor (without O_2_). It was found by DRIFTS that both linear (2114 cm^−1^, 1990 cm^−1^) and bridging (1928 cm^−1^) forms of coordinated CO were formed upon adsorption onto the catalyst surface. Moreover, the formation of CO_2_ was detected upon the interaction of the coordinated CO with oxygen. The kinetics of CO oxidation was studied at 18–38 °C at an atmospheric pressure for CO, O_2_, N_2_, and H_2_O (gas) mixtures in a flow reactor (steady state conditions).

## 1. Introduction

Carbon monoxide (CO) oxidation is among the simplest and most widely used oxidation reactions. Its mechanism is of both fundamental and practical interest. The fundamental interest in this reaction is related to gaining a deeper insight into the involvement of molecular oxygen in oxidation processes. The applied research in this area is aimed at improving the low-temperature CO oxidation catalysts that are presently employed in individual and collective protective equipment against carbon monoxide emitted into the atmosphere by fires and as a result of fuel combustion at industrial enterprises and in motor vehicle engines [1,2,3].

The oxidation of CO to CO_2_ by molecular oxygen is catalyzed by heterogeneous and homogeneous catalysts in the gas and liquid phases, respectively, and this reaction has been investigated in sufficient detail [1,2,3,4,5,6,7,8,9,10]. The mechanism of low-temperature carbon monoxide oxidation over supported metal complex catalysts has been investigated to a lesser extent. Most studies have been devoted to the mechanism of CO oxidation over PdCl_2_–CuCl_2_/support catalysts (support = alumina, silica, activated carbon, etc.) [1,2,3,11,12,13,14,15,16,17,18,19,20,21]. It was initially suggested that the mechanism of catalysis in this case is analogous to that of compositionally similar homogeneous systems used in the alkene oxidation reaction [22]. However, in the 1970s, kinetic studies made it clear that the carbon monoxide oxidation mechanisms are more diverse and complicated than the alkene oxidation mechanisms in similar systems. For example, low-temperature CO oxidation involving a homogeneous or heterogeneous catalytic system is characterized by an induction period attributed by most researchers to the generation of catalytically active sites of palladium in an oxidation state intermediate between 2+ and 0 [23]. Depending on the reaction conditions and partial pressures of carbon monoxide, oxygen, and water, the formal kinetic orders of the reaction with respect to CO, O_2_, and H_2_O vary over a wide range [2,10,11]. 

The following hypothetic mechanisms of heterogeneous catalytic CO oxidation have been discussed in the literature: (i) the formation of a liquid film on the support surface, which dissolves the active components; (ii) classical stepwise mechanisms, whereby palladium(II) oxidizes CO, copper(II) oxidizes the reduced form of palladium, and the resultant copper(I) is oxidized by oxygen; and (iii) conventional Langmuir–Hinshelwood heterogeneous catalytic mechanisms [2,3,10,11]. However, a mechanism fully consistent with all available experimental data and an adequate kinetic model remains elusive. This is due to insufficient knowledge on the chemical states of palladium and copper on the support surface during the process. This information, mainly related to the state of copper and palladium chlorides on the support surface, has appeared in the last 15–20 years owing to works by several research teams [10,11,12,13,14,15,16,17,18,19,20,21]. 

For most of the investigated PdCl_2_–CuCl_2_/support systems, copper exists in the form of Cu_2_Cl(OH)_3_ (paratacamite) and CuCl_2_·H_2_O crystalline nanophases according to X-ray absorption spectroscopy (XAS) and X-ray diffraction (XRD) [12,13,14,15,16]. Information on the state of palladium on the surface of a freshly prepared catalyst is more ambiguous. No palladium-containing crystalline phases on the catalyst surface have been identified. The palladium coordination environment initially consists of chlorine atoms but terminal and bridging carbonyl ligands emerge upon carbon monoxide exposure, as it was demonstrated by infra-red spectroscopy [10,11]. Recently, we monitored changes in the coordination environment of copper and palladium on going from solid PdCl_2_ and CuCl_2_·2H_2_O salts (used as reactants) through impregnation by aqueous solutions and finally to the metal-containing active sites on the surface of the freshly prepared nanocatalyst using scanning electron microscopy (SEM), X-ray diffraction (XRD), X-ray absorption spectroscopy (X-ray absorption near-edge structure (XANES), Extended X-ray absorption fine structure (EXAFS)) [24]. It was demonstrated that the state of palladium remains essentially the same in solid PdCl_2_, mixed palladium-copper(II) chloride solutions, and γ-Al_2_O_3_-supported catalysts; specifically, it remains in the oxidation state 2+ and in a square-planar coordination environment with neighboring chlorine atoms. No signatures of copper atoms in the local environment of palladium were found [24]. For the PdCl_2_–CuCl_2_/γ-Al_2_O_3_ catalyst, EXAFS data imply a slightly distorted square-planar chloride environment with one of the palladium–chlorine bonds longer than the three others. The atomic radial distribution curve for the catalyst shows weak long-range order peaks at 3–4 Å, which are better described as aluminum atoms rather than Pd–Pd or Pd–Cu atomic pairs. This is indicative of the formation of Pd–Cl–Al bridging bonds at ion-exchange sites of the support upon chemisorption [24]. 

As for the state of copper, our EXAFS and XRD data [24] agree with other research teams [12,13,14,15,16,17,18,19,20]: the cold impregnation of the support gives rise to a crystalline Cu_2_Cl(OH)_3_ nanophase with the paratacamite structure. The formation of this nanophase is possibly favored by basic sites of alumina.

Herewith, we extend the study of the mechanism of low-temperature carbon monoxide oxidation reporting on changes in the states of copper and palladium in the CuCl_2_/γ-Al_2_O_3_ and PdCl_2_–CuCl_2_/γ-Al_2_O_3_ nanocatalysts under reactive conditions in the atmosphere of CO, O_2_, and H_2_O vapor in various combinations at room temperature and discussing relevant kinetic measurements.

## 2. Materials and Methods 

Three samples were characterized by instrumental techniques. Sample 1 was γ-Al_2_O_3_ (0.5–1 mm size fraction with a BET (Brunauer-Emmett-Teller) surface area of 219 m^2^/g). Samples 2, 3 and 4 were obtained by cold impregnation of γ-Al_2_O_3_ with aqueous solutions of CuCl_2_·2H_2_O (sample 2) and PdCl_2_ + CuCl_2_·2H_2_O (samples 3, 4, catalysts) using the same alumina fraction as in the sample 1 (reference). The samples dried to the constant weight at room temperature contained 3.5 wt % (samples 2 and 3) and 17.5 wt % of copper (sample 4) and 1.5 wt % of palladium (samples 3 and 4, catalysts) with respect to γ-Al_2_O_3_ support.

Samples 1,3,4 were studied by powder X-ray diffraction with the use of synchrotron radiation with the same alumina size fraction. Fuji Film Imaging Plate photosensitive plates (Fuji Photo Film Co., Ltd., Minami-Ashigara-Shi, Japan) served as a two-dimensional detector; the diffraction patterns were digitized with the aid of a Fuji Film BAS-5000 scanner (Fuji Photo Film Co., Ltd., Tokyo, Japan) with a space step of 100 μm. The radiation wavelength was 0.46416 Å, the distance between the sample and the detector was 230 mm, and the exposure time was 30 min. The study was carried out at room temperature. The two-dimensional diffraction patterns were primarily processed using the Fit2D program (Grenoble, France) [25].

X-ray absorption near-edge structure (XANES) and extended X-ray absorption fine structure (EXAFS) measurements were performed at the Structural Materials Science beamline of the Kurchatov Synchrotron Radiation Center [26]. A special cell designed for catalysis-oriented in situ studies has been used [27].

Diffuse reflectance infrared Fourier transform spectroscopy (DRIFTS) studies were carried out in the 6000–400 cm^−1^ range at 4 cm^−1^ steps using a Nicolet Protégé 460 spectrometer equipped with a diffuse-reflectance attachment developed at the N. D. Zelinsky Institute of Organic Chemistry [28]. Granular samples were placed in a tube supplied with a KBr window and a glass two-way vacuum valve. CaF_2_ powder was used as a reference substance. Before recording the spectra, samples 1–3 were evacuated at room temperature to a residual pressure of 8 × 10^−2^ Torr for 4 h to remove physically adsorbed water. In order to elucidate the electronic states of palladium and copper, CO adsorbed at an equilibrium pressure of 20 Torr was used as probe molecule. Furthermore, sample 3 (catalyst) was examined under reactive (CO oxidation) conditions. CO was introduced into the tube at room temperature to an equilibrium pressure of 30 Torr, and the system was kept under these conditions for 50 min. Thereafter, air was admitted into the tube up to the atmospheric pressure. DRIFTS measurements were taken in a narrow wavenumber range from 2450 to 1700 cm^−1^.

The kinetics of low-temperature carbon monoxide oxidation were studied in a temperature-controlled flow reactor at a reactant conversion not exceeding 15% so that the reactor can be regarded as a gradientless (differential) reactor. It required the linear velocity of a gas flow to be 5 cm/s or higher (30,000 h^−1^ or more). The gas composition at the inlet and outlet of the reactor was determined by gas chromatography using 3 m × 3 mm columns filled with activated carbon AR-3 (0.25–0.5 mm fraction) and zeolite 13× (0.25–0.5 mm fraction), a thermal-conductivity detector, and argon as a carrier gas. The gas temperature and the water vapor content in the outlet gas were monitored using an IVTM-7-03-03-01 thermo-hygrometer (RF Specifications TU 4311-001-29359805-01, Moscow, Russia). The catalyst temperature was measured using an electronic thermometer with a sensor placed inside the catalyst bed. 

During kinetic tests with sample 3 (PdCl_2_–CuCl_2_/γ-Al_2_O_3_ containing 1.5 wt % Pd, 3.5 wt % Cu) in the flow reactor at a high CO concentration (~6 vol %) in the inlet gas mixture (it was necessary to operate in the low CO conversion mode), the activity of the catalyst dropped drastically, and the sample changed its color from light brown to dark gray. This was apparently due to progressive irreversible reduction of palladium(II) to inactive palladium(0). In order to suppress this process and to enhance the stability of the catalyst under the CO oxidation conditions, it was necessary to increase the copper(II) content in the catalyst up to 17.5 wt % at a constant palladium content (sample 4).

## 3. Results and Discussion

**Figure 1:**
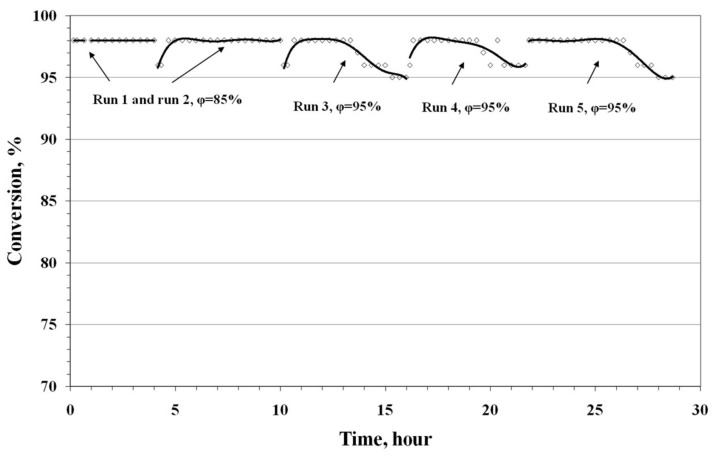
Results of PdCl_2_–CuCl_2_/γ-Al_2_O_3_ catalyst testing in the flow reactor in CO oxidation with O_2_ of air (100 mg/m^3^ CO in air, gas hourly space velocity (GHSV) = 12,000 h^−1^, 22 ± 0.5 °C, humidity(ϕ) is shown in Figure 1).

The PdCl_2_–CuCl_2_/γ-Al_2_O_3_ catalyst in different modifications was designed to be active at near room temperatures (15–70 °C) and in a wide range of humidity (20–95%) for use as the main component in masks and collective protective equipment against carbon monoxide in air (see, for example, Rus. Pat. 2267354, 2006). The results of testing this catalyst (1.5 wt % Pd, 3.5 wt % Cu) are shown in Figure 1 (22 °C, 100 mg/m^3^ CO, humidity 85–95%). The activity of the catalyst increases at the beginning of every run (a more active catalyst formation), then the period of the constant activity is observed. The activity of the catalyst decreases after a few hours under humidity 95%. It seems that the catalyst deactivation is connected with reduction of the active form to inactive palladium (0). The catalyst contacted with air during a few hours between the runs, and after that it was active again.

### 3.1. X-ray Diffraction Study

**Figure 2:**
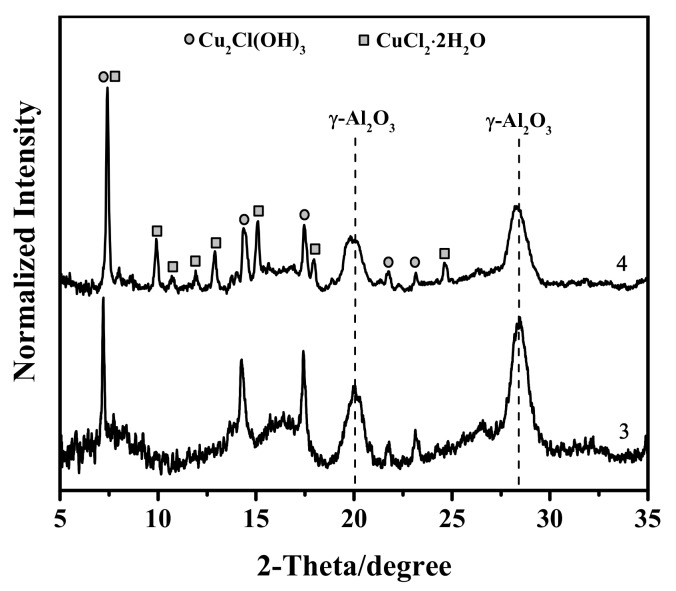
X-ray diffraction patterns of catalysts 3 (PdCl_2_–CuCl_2_/γ-Al_2_O_3_, 3.5 wt % Cu, 1.5 wt % Pd) and **4** (PdCl_2_–CuCl_2_/γ-Al_2_O_3_, 17.5 wt % Cu, 1.5 wt % Pd).

The X-ray diffraction pattern of sample 4 showed reflections from the Cu_2_Cl(OH)_3_ nanophase and copper(II) chloride dihydrate (Figure 2), whereas only Cu_2_Cl(OH)_3_ is present in sample 3. We believe that no dramatic changes in the reaction mechanism take place as the copper(II) chloride content in the catalyst increased. The only role of the additional amount of copper is to prevent the formation of the inactive phase of metallic palladium. Thus, in our kinetic experiments, the copper content in the catalyst was increased to 17.5 wt % and the catalyst granule size was kept within 0.5–1 mm. The experimental conditions were set so that the reaction proceeded under the kinetic control.

### 3.2. In Situ XAS Examination of the Catalyst

**Figure 3:**
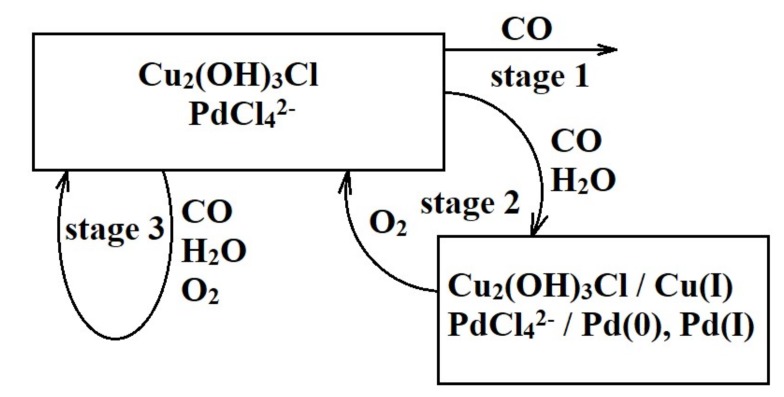
Scheme of in situ X-ray absorption spectroscopy (XAS) experiments.

In situ X-ray absorption spectroscopy was used to elucidate the structural changes occurring with the active sites of the catalyst in the presence of reactive species in the gas atmosphere. The sequence of experiments is schematically illustrated in Figure 3. 

At the first stage, the catalyst was treated with carbon monoxide (0.02 vol % in dry nitrogen) for about 12 h and no significant changes in the Pd*K*-edge and Cu *K*-edge XANES/EXAFS spectra were observed. Therefore, it may be concluded that neither carbon monoxide oxidation nor Pd^2+^/Cu^2+^ reduction occurs under these conditions at all or proceeds very slowly. 

**Figure 4:**
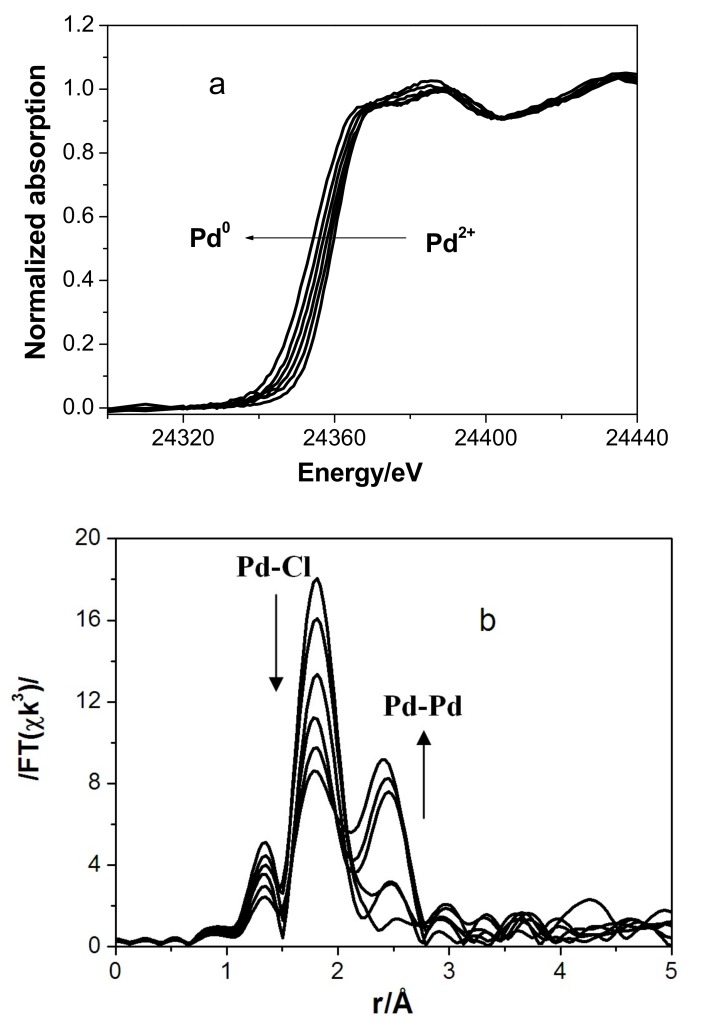
Second stage of in situ XAS experiments (Pd*K*-edge), room temperature. Temporal evolution of (**a**) XANES and (**b**) EXAFS spectra, sample 3. The atomic radial distribution curves were obtained by Fourier transform of EXAFS spectra.

At the second stage of the experiment, traces of water vapor were added to the CO atmosphere. Carbon monoxide and traces of water vapor reduce Cu^2+^ to Cu^+^ and Pd^2+^ to Pd^+^, Pd^0^. Under these conditions, Pd*K*-edge XANES/EXAFS spectra manifested profound changes: a progressive shift in the edge position was observed in XANES due to Pd^2+^→Pd^0^ reduction (Figure 4a). Meanwhile, Fourier transforms of EXAFS spectra demonstrated emergence and growth of the Pd–Pd peak due to simultaneous decreasing Pd–Cl peak (Figure 4b).

**Figure 5:**
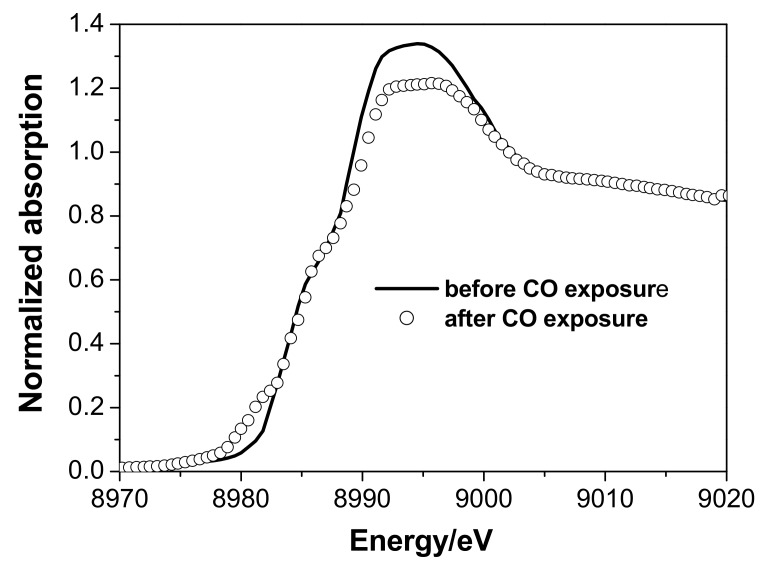
Second stage of in-situ XAS experiment (Cu *K*-edge). XANES spectrum of the catalyst 3 at the beginning and at the end of the CO exposure, room temperature.

The Cu *K*-edge XANES and EXAFS spectra indicated a partial reduction of Cu(II) to Cu(I) with a general retention of the paratacamite structure subject to some disordering (Figure 5).

These results suggest that the stoichiometric carbon monoxide oxidation occurs, and this process is associated with palladium(II) and copper(II) reduction to Pd(0) and Cu(I), respectively, in the presence of CO and H_2_O but in the absence of oxygen. Remarkably, the chemical state of Pd and Cu in the catalyst is completely restored after exposure to air.

**Figure 6:**
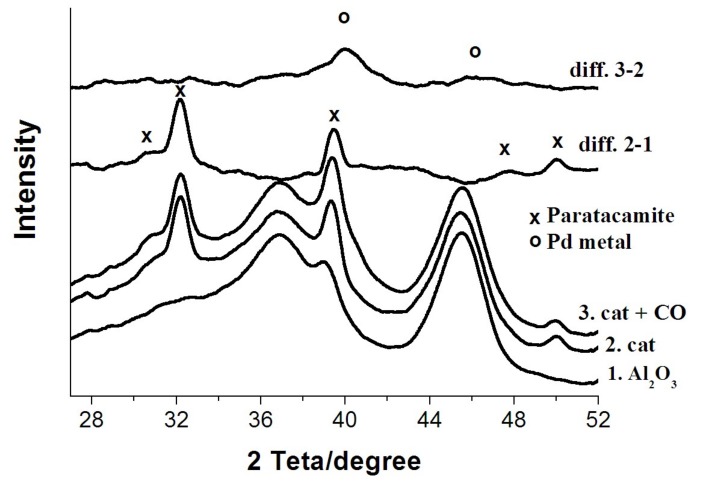
Results of in situ X-ray diffraction, room temperature, samples 1, 2, 3.

X-ray diffraction patterns measured in situ in parallel with X-ray absorption fine structure (XAFS) spectra give further insight into the chemical processes (Figure 6).

Due to the low concentration of active Pd and Cu components supported on Al_2_O_3_, some changes in the diffraction patterns become apparent only in difference curves. In particular, the difference curve “freshly prepared catalyst—γ-Al_2_O_3_ support”, i.e., 2-1 in Figure 6, confirms that the deposition of CuCl_2_ and PdCl_2_ onto γ-Al_2_O_3_ yields the paratacamite nanophase Cu_2_(OH)_3_Cl in agreement with earlier studies [12,13,14,15,16,17,18,19,20,24]. The palladium-containing phase is X-ray-amorphous and, according to the literature [15,19,24], represents tetrachloropalladate [PdCl_4_]^2−^ clusters chemisorbed on γ-Al_2_O_3_. The treatment of the catalyst with CO in the presence of moisture as evidenced by the difference diffraction curve 3-2 yields a nanosized phase of metallic palladium, whereas the paratacamite phase remains essentially intact. 

Even more detailed information about the products of the interaction between carbon monoxide and the catalyst and about their transformations in contact with air has been obtained by the DRIFTS method (see below).

At the third stage of the experiment, the catalyst was placed into the three-component reactive atmosphere mimicking the real catalytic process, i.e., CO + H_2_O + O_2_. Under these conditions, no spectral changes were detected within several hours. This is probably due to the fact that a dynamical equilibrium between reduced and oxidized forms of active species within the catalyst is established with the latter ones dominating: reduced copper(I) is rapidly oxidized by oxygen to the oxidation state 2+ and converts palladium(0) into its oxidized state, thus ensuring continuous regeneration of the catalyst. This should correspond to a high activity of the catalyst.

### 3.3. In Situ Diffuse Reflectance Infrared Fourier Transform Spectroscopy DRIFTS Examination of the Catalyst

**Figure 7:**
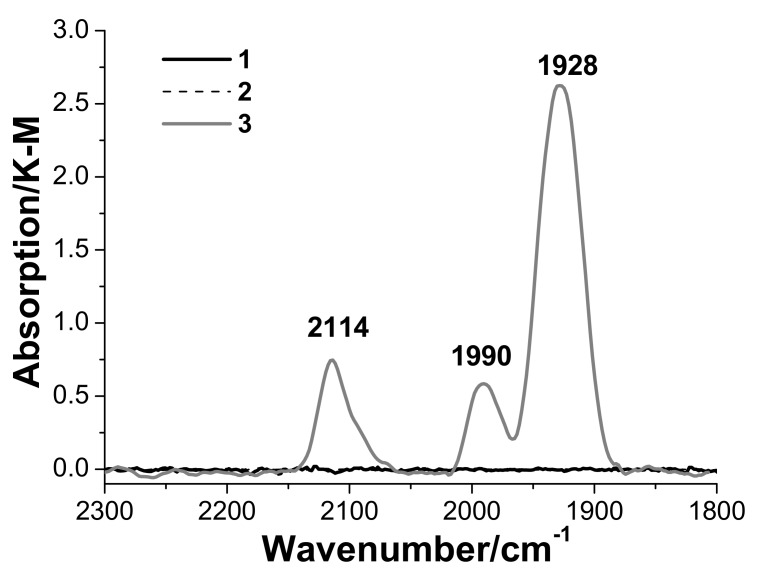
DRIFT spectra of samples 1 (Al_2_O_3_), 2 (CuCl_2_/Al_2_O_3_) and 3 (PdCl_2_–CuCl_2_/γ-Al_2_O_3_) at an equilibrium CO pressure of 20 Torr and room temperature (the intensity scale is expressed in Kubelka-Munk units).

The diffuse-reflectance IR spectra of samples 1 (Al_2_O_3_) and 2 (CuCl_2_/Al_2_O_3_) exposed to carbon monoxide at room temperature show no bands characteristic of stretching vibrations of the C≡O bond in the CO molecule (Figure 7). 

At the same time, the spectrum of sample 3 (CuCl_2_−PdCl_2_/Al_2_O_3_) recorded under the same conditions displays three bands, namely, a strong band at 1928 cm^−1^ and two weaker bands at 1990 and 2114 cm^−1^.

According to the literature data [11,29,30,31], vibrational frequencies below 1800 cm^−1^ are due to Pd(II) complexes with an inserted CO group (XPdC(O)Y), whose state is similar to the state of µ-carbonyl groups in organic compounds. Vibrational frequencies in the 1800–2000 cm^−1^ range correspond to Pd(I) complexes with a bridging CO group, while vibrational frequencies above 2000 cm^−1^ are due to Pd(II) complexes with a terminal CO group. The presence of CO molecules coordinated as bridges in Pd^0^ clusters is manifested in the 1800–1890 cm^−1^ range. Vibrational frequencies of CO groups on the surface of metallic palladium can be observed in the 1800–1880, 1800–2000, and 2050–2120 cm^−1^ ranges, which are characteristic of µ^3−^, µ^2−^, and terminal CO groups, respectively [11]. No spectroscopic data are presently available for structurally characterized complexes of Pd(I) with terminal CO groups and for those of Pd(II) with bridging CO groups. Cu(I) complexes with terminal CO groups are characterized by vibrational frequencies in the 2050–2120 cm^−1^ range. There is no reliable information on Cu(II)–CO complexes in the available literature. 

The IR spectroscopic data available in the literature on adsorbed CO do not allow the band at 2114 cm^−1^ in the spectrum of sample 3 to be unambiguously assigned. This band may be due to the terminal form of CO adsorbed on coordinatively unsaturated Pd(II) atoms, Pd^0^, or Cu(I).

We believe that the band at 2114 cm^−1^ observed in the IR spectrum of sample 3 is due to the terminal form of CO adsorbed on Cu(I) species. The bands at 1990 and 1928 cm^−1^ characterize the adsorption of CO in the bridging form on Pd(0) and/or Pd(I). It seems most likely that the moderate-intensity band at 1990 cm^−1^ is related to symmetric vibrations of bridging CO groups on Pd(I), while the strong band at 1928 cm^−1^ is due to antisymmetric vibrations, similar to the case of the dimeric anion [Pd_2_(CO)_2_Cl_4_]^2−^ (characterized by the bands at 1973 and 1922 cm^−1^) [32]. Reduced palladium and copper species are formed via the Pd(II) and Cu(II) reduction by carbon monoxide in the presence of a small amount of water that is retained on the surface of sample 3 even after its treatment in a vacuum. According to Pd*K*-edge (Figure 4a) and Cu *K*-edge XANES data (Figure 5), an appreciable fraction of copper and palladium remains in the oxidation state +2 even after a prolonged treatment of the catalyst with CO. Note that the amount of carbon monoxide introduced into the tube containing the sample for DRIFTS measurements is insufficient for the complete reduction of Pd(II) to Pd(I)/Pd(0) and of Cu(II) to Cu(I). We were unable to detect carbon dioxide (CO oxidation product) coordinated on the surface or present in the gas phase. This is probably explained by a very small amount of CO_2_ formed. The absence of absorption bands of the coordinated CO group in the IR spectra of CuCl_2_/γ-Al_2_O_3_ exposed to CO, as well as the results of special experiments performed in order to check for the catalytic activity of this sample, suggest that copper(II) present as Cu_2_Cl(OH)_3_ with the paratacamite structure on the γ-Al_2_O_3_ surface does not oxidize CO to CO_2_ to any considerable extent in the absence of palladium under the conditions studied [24]. Thus, palladium promotes the oxidation of carbon monoxide by copper(II). 

**Figure 8:**
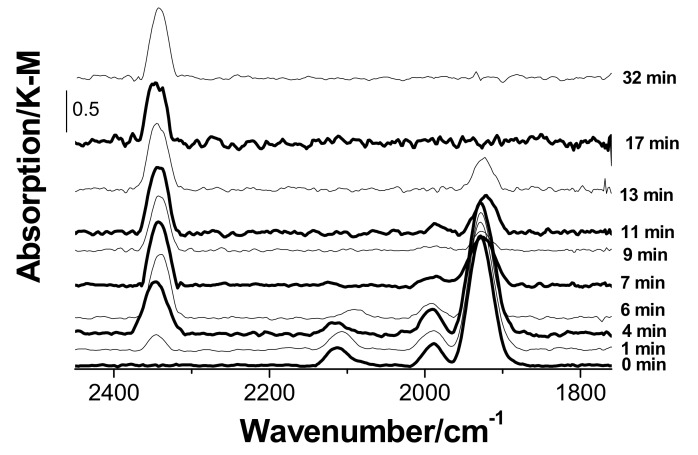
DRIFT spectra of sample 3 (PdCl_2_–CuCl_2_/γ-Al_2_O_3_). Conversion of coordinated CO involving atmospheric oxygen.

The IR spectrum presented in Figure 7 remains unchanged for 50 min. Exposure of the sample to air causes gradual changes as illustrated in Figure 8. First, a band at 2346 cm^−1^ that is characteristic of adsorbed CO_2_ [30] appears already in 1 min. The intensity of this band increases for 7 min and then remains unchanged until the 32nd minute, while the bands of CO groups are gradually vanishing. This behavior suggests that oxygen is directly involved in the formation of CO_2_ from CO.

Note that the above results are fairly consistent with earlier [11] and more recent [33] data for catalysts that are similar in the composition and preparation procedure to our catalyst; however, there are significant distinctions. For example, according to Shen et al. [33], the most prominent absorption bands in the diffuse-reflectance IR spectra of a similar catalyst exposed to carbon monoxide are the bands at 2162 and 2126 cm^−1^ assigned by Shen et al. [33] to the CO groups adsorbed on Pd(II) and Cu(I), respectively. In the spectra of catalyst 3 recorded by us under similar conditions (Figure 7 and Figure 8), the band at 2162 cm^−1^ is missing, but there is a band at 2114 cm^−1^ assigned to the terminal CO group on Cu(I). The most intense band in our spectrum is observed at 1928 cm^−1^ attributed to the bridging CO group coordinated to Pd(I). In the spectrum of a similar catalyst examined by Shen et al. [33], this band is missing together with the band at 1990 cm^−1^, which was also assigned by us to the bridging CO group coordinated to Pd(I) or Pd(0). The bands with close maxima were observed in the spectra of catalysts prepared by cold impregnation using aqueous ammonia [33] and in the spectrum of a similar catalyst reported by Choi and Vannice [11]. These authors additionally observed a weak band at 2158 cm^−1^ (at a low partial pressure of CO or under reactive conditions) [11], which was assigned to terminal CO groups on Pd(II), and a shoulder at 2080 cm^−1^ attributed to vibrations of terminal CO groups on Pd^0^. Unlike Choi and Vannice [11], we did not observe the reduction of Cu(II) in CuCl_2_/γ-Al_2_O_3_ by carbon monoxide under mild conditions. Note that Shen et al. [33] observed CO_2_ formation in the gas phase (indicated by the appearance of a band at 2300–2400 cm^−1^), when a CO + O_2_ or CO + O_2_ + H_2_O mixture contacted with the catalyst. 

The most important results of our spectroscopic study are as follows.

(1) There is no evidence of a direct copper–palladium interaction (such as the formation of mixed complexes) on the surface of a freshly prepared nanocatalyst;
(2) When the catalyst is exposed to CO and water vapor (in the absence of oxygen), palladium(II) and copper(II) undergo slow reduction to yield Pd(I), Pd(0), and Cu(I). In the absence of palladium, copper in the form of hydrated copper(II) chloride and paratacamite nanophases on the support surface does not interact noticeably with CO and H_2_O at room temperature;
(3) Palladium and copper carbonyl complexes with bridging and terminal carbonyl groups are formed as products of the interaction of the catalyst with CO and H_2_O. No CO_2_ was observed among the products;
(4) Bringing the catalyst containing adsorbed CO into contact with oxygen causes rapid decomposition of the carbonyl complexes and the formation of carbon dioxide. Apparently, oxygen is directly involved in carbon dioxide formation steps;
(5) The methods used in this study indicated no palladium(II) or copper(II) reduction under the conditions of catalytic carbon monoxide oxidation;
(6) The simultaneous presence of optimum amounts of oxygen and water vapor in the reaction mixture is a necessary condition for the active and steady operation of the low-temperature CO oxidation over the nanocatalyst PdCl_2_–CuCl_2_/γ-Al_2_O_3_.

### 3.4. Results of Preliminary Kinetic Studies 

**Figure 9:**
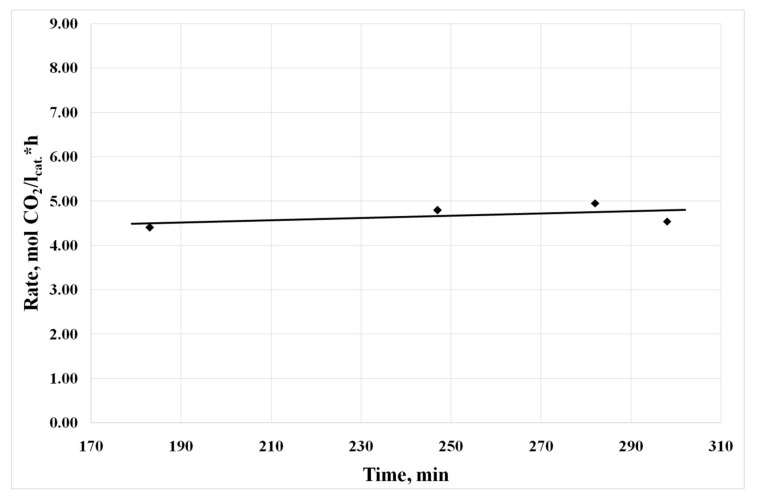
Dependence of the reaction rate on the time of the experiment.

Each kinetic experiment was carried out with a fresh sample of catalyst 4 (PdCl_2_–CuCl_2_/γ-Al_2_O_3_, 1.5 wt % Pd, 17.5 wt % Cu). The reaction rate was constant under definite conditions in every kinetic experiment (see, for example, Figure 9).

Conditions: GHSV = 30,000 h^−1^, inlet gas composition (vol %): CO—6.24, O_2_—18.47%, N_2_—73.75, H_2_O—1.54 (absolute humidity—11 g/m^3^), catalyst temperature—27.0 ± 0.2 °C.

**Figure 10:**
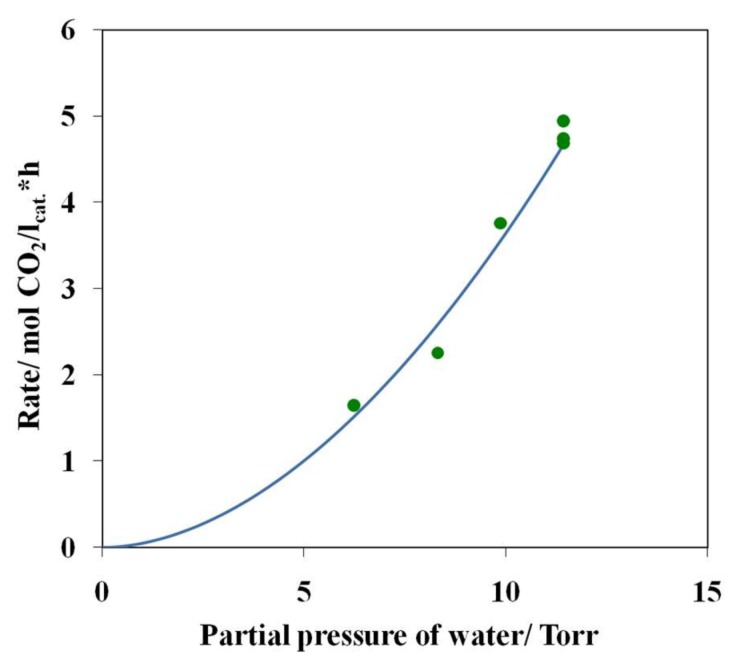
Rate of CO_2_ formation as a function of partial pressure of water at 27 °C and at the constant partial pressures of oxygen (142.9 Torr) and carbon monoxide (45.7 Torr), sample 4.

**Figure 11:**
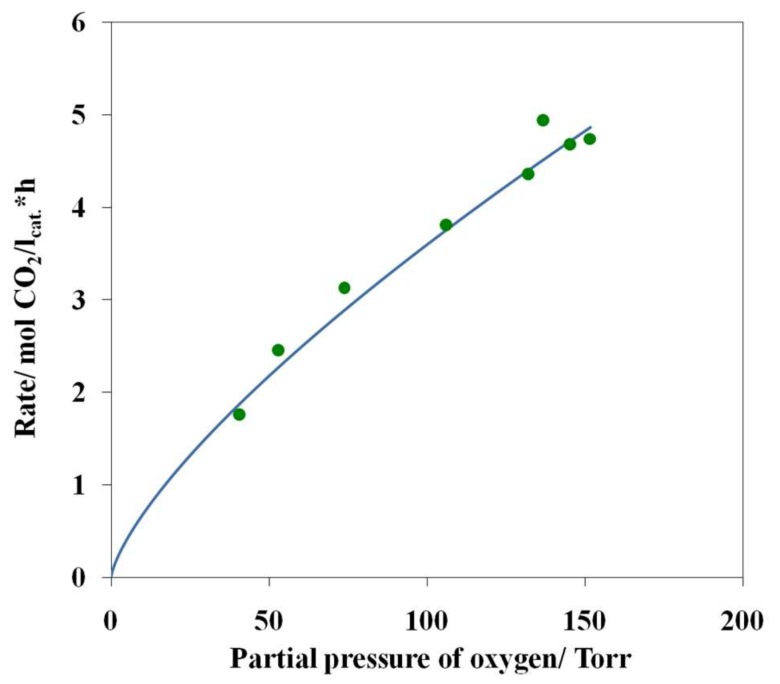
The rate of CO_2_ formation as a function of the partial pressure of oxygen at 27 °C and at the constant partial pressures of water (11.4 Torr) and carbon monoxide (45.7 Torr), sample 4.

**Figure 12:**
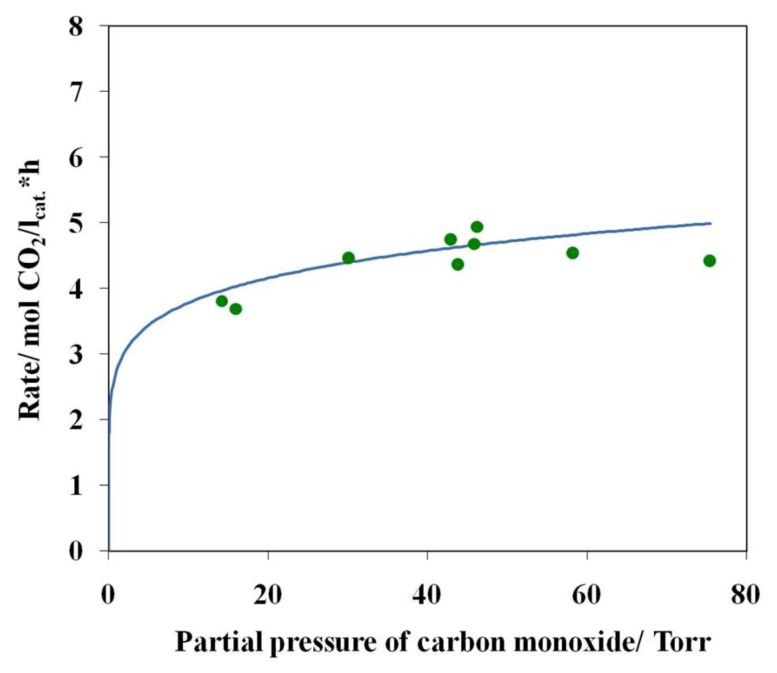
The carbon dioxide formation rate as a function of the carbon monoxide partial pressure at 27 °C and at the constant partial pressures of water (11.4 Torr) and oxygen (142.9 Torr), sample 4.

By performing a single-factor experiment at 27 °C, we have determined the partial dependences of the carbon dioxide formation rate on the partial pressures of water, oxygen, and carbon monoxide (Figure 10, Figure 11 and Figure 12).

In order to check for an effect of the carbon dioxide partial pressure, we added carbon dioxide to the initial gas mixture. The introduction of a 100-fold excess of carbon dioxide (with respect to that produced in the catalytic reaction) did not affect the carbon monoxide conversion, which remained the same as in the experiment conducted under the identical conditions but in the absence of carbon dioxide. 

Analysis of these data demonstrated that the formal order of the reaction with respect to partial pressures of water is ~1.86 and the order with respect to partial pressure of oxygen is ~0.72 (Figure 10 and Figure 11). The order of the reaction with respect to the partial pressure of carbon monoxide is ~0.14 (Figure 12).

Thus, the above dependences can be satisfactorily approximated by the following power-law equation:

*R*_CO_2__ = *k P*_CO_^0.14^*P*_H_2___O_^1.86^*P*_O_2__^0.72^ (1)

This equation was used for determining the apparent “activation energy” under the assumption of the constant reaction orders with respect to reactants over the tested temperature range. The temperature was varied in the 20–38 °C range while maintaining the constant partial pressures of water (11.4 Torr), oxygen (142.9 Torr), and carbon monoxide (45.7 Torr).

**Figure 13:**
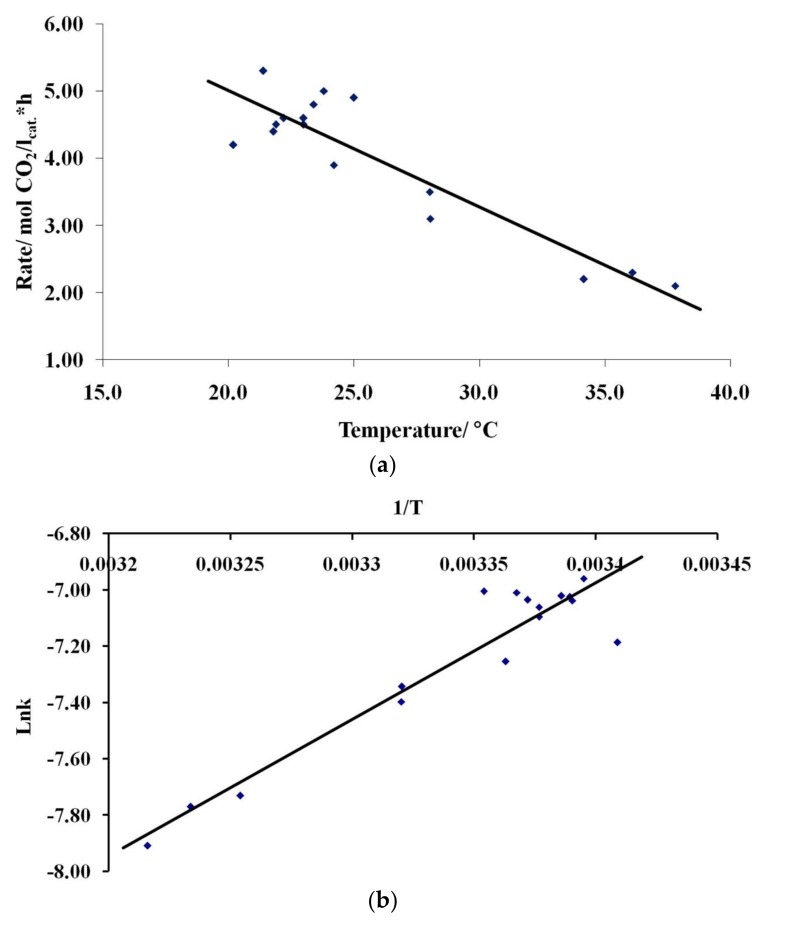
The temperature dependences of the reaction rate as the rate-temperature (**a**) and ln k − 1/T (**b**) plots.

Figure 13a shows the dependence of the carbon dioxide formation rate on the reaction temperature with all other parameters kept constant and the Arrhenius plot (Figure 13b). The oxidation rate of carbon monoxide decreases with increasing temperature. The apparent activation energy of the catalytic reaction is about −40 kJ/mol, i.e., a negative value, in the given temperature range under the assumption that the orders of the reaction remain constant as temperature is varied.

The finding that the order of the reaction with respect to the CO partial pressure is close to zero confirms that carbon monoxide may be involved in the formation of active sites such as Pd_2_(CO)_2_^2+^ but it does not participate in rate-limiting stages. 

The high order of the reaction on the partial pressure of water is probably due to the adsorption of two water molecules on an active site and the participation of these molecules in the formation of carbon dioxide. The exothermicity of water adsorption most likely accounts for the observed negative apparent activation energy of the process. 

The nearly first order of the reaction on the partial pressure of oxygen is consistent with the observed rapid decomposition of palladium carbonyl complexes after contact with oxygen and can be considered as an evidence of the direct involvement of oxygen in the CO_2_ formation. 

Unfortunately, the above data do not clarify unambiguously the role of copper in the mechanism of the whole process. If crystalline Cu_2_Cl(OH)_3_ on the support surface oxidizes Pd^0^ or other forms of reduced palladium, e.g., hydride complex, then copper(II) hydroxychloride has to be in close contact with the palladium compound. However, the experimental data obtained so far do not confirm or support the formation of mixed complexes of palladium and copper in the nanocatalyst. Note that, if such palladium–copper mixed complexes are formed only in situ and involve only a minor fraction of the total amount of palladium and copper, these complexes are very difficult to be detected by the experimental techniques used in this study. In principle, the formation of mixed complexes is quite likely. Indeed, several palladium(II)- and copper(II)-containing complexes have been synthesized in organic solvents, in particular [(PdCl_2_)_2_CuCl_2_L_2_] [34] and [Pd_6_Cu_2_Cl_12_O_4_L_4_] [35], where L is a coordinated molecule of an organic solvent (DMF or HMPA). 

Another function of copper could be the activation of molecular oxygen by coordination to Cu(I) followed by the interaction of the resulting complex with a palladium hydroxycarbonyl complex:

(2) $$-{\overset{|}{\underset{|}{\mathrm{Pd}}}}^{\mathrm{II}}(\mathrm{COOH})+-Cu^{I}(O_{2})\overset{\;H^{+}\;}{\to }\mathrm{Pd}(\mathrm{II})+\mathrm{Cu}(I)+H_{2}O+CO_{2}$$

**Figure 14:**
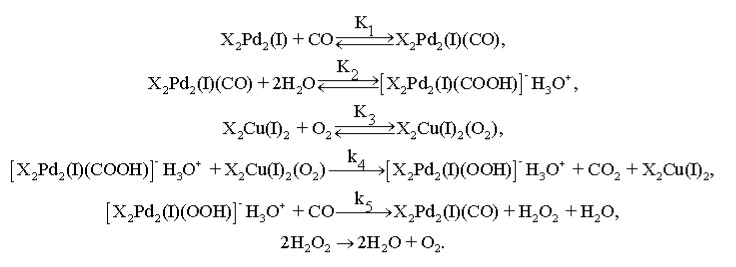
The simplest mechanism of low-temperature CO oxidation with O_2_ on a PdCl_2_–CuCl_2_/γ-Al_2_O_3_ catalyst.

However, the implementation of this function would need either the presence of Cu(I) and Pd(II) in a single complex or their interaction on the support surface. The simplest mechanism consistent with all the obtained data is shown in Figure 14.

The negative value of the apparent activation energy of the reaction examined is in agreement with the high order of the reaction on the partial pressure of water in the above equation for the rate of carbon dioxide formation, indicating that water plays a key role in the carbon monoxide oxidation. A detailed kinetic study of this process and discrimination of hypotheses concerning the reaction mechanism will be the subject of a forthcoming report.

## 4. Conclusions

The main results of our study are as follows. Palladium at the surface of the PdCl_2_–CuCl_2_/γ-Al_2_O_3_ nanocatalyst is present in the form of PdCl_4_^2−^-containing amorphous particles, while copper exists as crystal nanophases with the crystal structure of paratacamite (Cu_2_(OH)_3_Cl) and cuprous chloride(II) (CuCl_2_·2H_2_O). The interaction with CO and water vapor results in carbonyl complexes, which are intermediates in the carbon dioxide formation. Carbon dioxide is formed upon the cleavage of the palladium carbonyl complexes in the presence of preliminarily coordinated water and copper(I)-activated oxygen.

These data agree well with those obtained in early kinetic studies. A negative value of the apparent activation energy and a high order found in the equation describing the dependence of the carbon dioxide formation rate on the water partial pressure may be related to exothermic adsorption stages of water molecules involved in the CO_2_ formation. The high order with respect to the oxygen partial pressure agrees with the assumption that the copper(I)-activated oxygen is involved in the carbon dioxide formation.

These hypotheses need to be verified by systemic studies of kinetic regularities. The results of these studies will be published later elsewhere.

Evidently, the proper choice of the active metal, especially the most active state among the available forms of this metal, is crucial for designing the most efficient CO oxidation catalysts [36]. Of particular attention are the hybrid organic–inorganic materials that promise an advantageous combination of structural and porous characteristics [37,38,39,40].
